# Trajectory‐specific motion‐induced electromotive force assessment of pacemaker‐carrying workers in MRI environments

**DOI:** 10.1002/mp.70524

**Published:** 2026-07-16

**Authors:** Valentina Hartwig, Giuseppe Acri, Giovanni Calcagnini, Cecilia Vivarelli, Rosaria Falsaperla, Eugenio Mattei

**Affiliations:** ^1^ Institute of Clinical Physiology CNR Pisa Italy; ^2^ Dipartimento di Scienze Biomediche, Odontoiatriche e Delle Immagini Morfologiche e Funzionali Università Degli Studi di Messina Messina Italy; ^3^ Department of Cardiovascular, Endocrine‐metabolic Diseases and Ageing ISS – Italian National Institute of Health Rome Italy; ^4^ National Center for Artificial Intelligence and Innovative Health Technologies ISS – Italian National Institute of Health Rome Italy; ^5^ Department of Occupational and Environmental Medicine Epidemiology and Hygiene, Italian Workers' Compensation Authority (INAIL) Monteporzio Catone (Rome) Italy

**Keywords:** electromagnetic interference, fringe field, implantable/cardioverter defibrillator, magnetic resonance, motion‐induced electromotive force, MRI, pacemaker, static magnetic field

## Abstract

**Purpose:**

Motion of individuals carrying active implantable medical devices (AIMDs), such as pacemakers (PMs) and implantable cardioverter defibrillators (ICDs), within the spatially varying static magnetic field in and around magnetic resonance imaging (MRI) systems can induce electromotive forces (EMFs) in conductive leads. This mechanism is not explicitly addressed in standard MRI safety assessments. This study presents a measurement‐based computational framework to estimate motion‐induced EMF under representative exposure conditions relevant to clinical practice.

**Methods:**

Measured maps of the static magnetic field components (*Bx*, *Bz*) in the fringe region of 3 and 7 T MRI systems were interpolated on regular grids. Spatial gradients were derived and used to compute motion‐induced EMF in an equivalent conductive loop representing a PM/ICD lead configuration. Three motion types were considered: linear motion along the scanner axis (*z*), linear motion transverse to the bore (*x*), and in‐place rotation of the loop. EMF was calculated using Faraday's law, assuming a loop area of 0.0225 m^2^, a reference linear velocity of 1 m/s, and an angular velocity of 1 rad/s. Spatial EMF distributions and time‐dependent waveforms were obtained for each motion scenario.

**Results:**

Induced EMF varied significantly with motion direction and spatial location. For linear motion, the highest values were observed near the bore entrance, where strong spatial field variations occur. At 3 T, maximum EMF reached 160 mV (*z*‐motion) and 114 mV (*x*‐motion), while at 7 T the corresponding values were 81 and 104 mV for the analyzed trajectories. Rotational motion produced lower and more localized EMF values, with maxima of 30 mV (3 T) and 35 mV (7 T). Despite the higher nominal field strength, the 7 T system did not consistently yield higher EMF, reflecting differences in spatial field gradients.

**Conclusions:**

Motion‐induced EMF in MRI fringe fields depends on local spatial field gradients and motion characteristics rather than nominal field strength alone. The proposed measurement‐based framework provides spatially resolved, scenario‐specific estimates of EMF and can be applied to different MRI systems given appropriate field maps. This approach complements existing safety assessments by addressing motion‐related exposure conditions not explicitly covered in standard evaluations.

## 1 INTRODUCTION

Active implantable medical devices (AIMDs), including cardiac pacemakers (PMs) and implantable cardioverter defibrillators (ICDs), are increasingly prevalent in both patient and healthcare worker populations. At the same time, the clinical use of magnetic resonance imaging (MRI) continues to expand, with the growing deployment of high‐ and ultra–high‐field systems (3 and 7 T). While MR‐conditional devices and dedicated safety protocols have significantly reduced risks for scanned patients,^1^, ^2^, ^3^ the broader electromagnetic environment of MRI facilities remains a concern for individuals carrying AIMDs, particularly in the fringe field region outside the bore.

Generally, procedures to assess the hazards for workers bearing AIMDs are covered by European Directive 2013/35/ EU, which sets out the minimum health and safety requirements regarding the exposure of workers to the risks arising from electromotive force (EMF) of the frequency 0–300 GHz.^4^ According to this Directive, workers with AIMDs are considered “workers at particular risk” for which the employer has the obligation to perform an individual EMF risk assessment.

Exposure limits and safety procedures in MRI environments are also defined by international standards and guidelines, including IEC 60601‐2‐33 for MRI systems,^5^ ISO 14708 for AIMDs,^6^ ASTM MRI safety standards,^7^, ^8^, ^9^ and ICNIRP exposure guidelines.^10^, ^11^, ^12^

Moreover, the non‐binding guide to good practice for implementing Directive 2013/35/EU^13^ gives us practical indications for the risk assessment of workers with AIMD, according to the approach described in the EN 50527 technical standards family.^14^, ^15^, ^16^

Most safety assessments for AIMDs in MRI environments focus on patients undergoing imaging, where radiofrequency (RF) heating, gradient‐induced voltages, and device malfunction are the primary concerns.^17^, ^18^, ^19^, ^20^ Today, most (PM/ICD) systems are labeled as MR‑conditional, meaning that, under the specific conditions defined by the manufacturer, the risk associated with undergoing an MRI examination can be considered acceptable for patients with these devices. However, these conditions are based on a risk assessment conducted for the patient during MRI scanning and cannot be extrapolated to other scenarios, such as individuals working inside an MRI environment. Indeed, less attention has been devoted to occupational exposure scenarios involving healthcare workers with implanted devices who may move within the static magnetic field gradients surrounding the scanner. In these regions, spatial variations of the static field can be substantial, especially near the bore entrance and in high‐field systems. Movement through such gradients produces time‐varying magnetic flux in conductive loops, including implanted lead systems, potentially inducing EMF. Motion‐induced EMF arises from the interaction between body or device motion and the spatial gradient of the static magnetic field (*∂B*/*∂x*, *∂B*/*∂z*). The resulting induced voltages depend on local field gradients, loop geometry, orientation, and motion dynamics (translation or rotation). For AIMDs, induced voltages at the device input stage may represent a potential interference mechanism, with implications for inappropriate sensing, pacing inhibition, or unintended stimulation.^21^, ^22^, ^23^ These effects are particularly relevant for operators and staff who may repeatedly approach the scanner bore, lean into the gantry, or move near the magnet during routine workflow. Given the complexity of performing in vivo measurements of EMF induced by movements in MRI environments, previous studies have addressed electromagnetic interference and induced voltages in cardiac implantable devices in MRI environments using both in vitro phantom‐based measurements and numerical modeling approaches. In particular, experimental assessments on PM systems exposed to MRI fields using conductive phantoms have been reported by Mattei et al.,^21^, ^22^, ^23^, ^24^, ^25^ while recent works have employed full‐wave numerical simulations to estimate induced quantities in implantable leads and devices under MRI exposure conditions.^20^

The progressive introduction of higher‐field MRI systems increases the importance of quantitatively characterizing fringe‐field gradients and associated motion‐induced EMF. Notably, nominal field strength alone does not fully determine risk, since the spatial distribution of the static field and its gradients depends on magnet design and shielding configuration. Therefore, site‐specific field mapping combined with physics‐based EMF estimation provides a practical framework for evaluating representative high‐exposure scenarios.

In this work, we present a computational framework for estimating motion‐induced EMF in PM lead loops using measured static field maps. The approach combines spatially resolved magnetic field data with trajectory‐dependent motion models, including linear walking paths and in‐place body rotations. The simulator supports interactive graphical selection of motion scenarios and enables comparative assessment across different MRI systems. The methodology is applied to two MRI environments (3 and 7 T) to quantify induced EMF values for representative high‐exposure scenarios and identify spatial risk regions associated with operator movement in the scanner fringe field.

## 2 METHODS

To assess the maximum time‐varying magnetic field exposure relevant to a PM/ICD, we estimated the EMF induced during typical operator movements in the non‐homogeneous static magnetic field environment surrounding an MRI scanner. Specifically, we considered three representative types of motion: linear translation along the *z*‐axis (scanner bore direction), linear translation along the *x*‐axis (lateral direction), and rotation of the torso by 180° about the vertical *y*‐axis, simulating a turning motion of the body.

The magnetic field data were provided as spatial distributions of the magnetic field components *Bx*(*x*,*z*) and *Bz*(*x*,*z*), defined on a horizontal plane parallel to the ground with 0.01 m spatial resolution. The field maps were extracted at a height corresponding to the approximate position of an implant (e.g., PM lead tip) in a standing adult.

High‐resolution two‐dimensional maps of the static magnetic field components *Bx*(*x*,*z*) and *Bz*(*x*,*z*), evaluated at thoracic height, were used as input to the computational framework. The PM lead was modeled as an equivalent rigid conductive loop consistent with established implant safety modeling approaches.^26^ The loop plane was assumed to be aligned with the subject frontal plane (*XY* plane) in the reference orientation, such that the loop normal is directed along the *Z* axis when the subject faces the scanner bore. In accordance with Annex L of ISO 14117, the induction area *A* was treated as an equivalent planar loop of 225 cm^2^, which represents the maximum achievable effective induction area once realistic anatomical constraints and non‑planar lead trajectories are accounted for, and is recommended by the standard as the appropriate worst‑case assumption for induced‑EMF calculations.

The induced EMF was computed from Faraday's law in its general form,

(1) $$\mathrm{EMF}=-\frac{d}{dt}\int_{S}\vec{B}\left(\vec{r}\right)\cdot \hat{n}\;dS$$

where *S* is the loop surface and $\hat{n}$ its unit normal. For a rigid loop of constant area undergoing motion in a static but spatially non‐uniform magnetic field, the time variation of magnetic flux arises from spatial field gradients and/or time‐varying loop orientation.

To obtain tractable expressions, the magnetic field was assumed locally uniform over the loop surface. Under this condition, the flux integral reduces to the product of loop area and the magnetic field component normal to the loop plane. The magnetic field maps were acquired on a horizontal plane at approximately the bore height, corresponding to the vertical symmetry plane of the MRI magnet. Thus, they represent a region where the static magnetic field magnitude is close to its maximum and varies only weakly along the vertical direction. Given the effective loop radius (∼8–9 cm) and the characteristic spatial scales of the MRI fringe‐field gradients, the magnetic field can therefore be reasonably approximated by its value at the loop center. The resulting uncertainty on the estimated EMF is conservatively below 10%–20% even in high‐gradient regions near the bore entrance (∼3 T/m),^27^ providing a conservative safety margin suitable for screening‐level risk assessment.

Spatial derivatives of the magnetic field components were computed on the measurement grid using central finite differences. Measured magnetic field maps acquired on a 0.1 m grid were interpolated to a 0.01 m grid using cubic interpolation prior to spatial derivative computation. This step improves the numerical stability of gradient estimation without introducing additional physical assumptions or artificial smoothing. Additional filtering was tested but showed negligible impact on EMF estimates and was therefore not applied in the final analysis. Finally, continuous field values and gradients along motion trajectories were obtained using gridded interpolation.

For linear motion at constant velocity *v*, the EMF reduces to the directional derivative of the magnetic field component normal to the loop plane along the direction of motion. When motion occurs along scanner bore direction, the loop normal is aligned with the *z*‐axis and the relevant component is *Bz*, yielding

(2) $$\mathrm{EM}F_{z}\;\left(t\right)=-Av\frac{\partial B_{z}}{\partial z}$$

where *A* is the induction area formed by the implant (225 cm^2^ according to ISO 14117 standard^26^). When motion occurs along the lateral direction, the loop normal is aligned with the *x*‐axis and the effective flux variation is governed by the spatial variation of *Bx*, giving

(3) $$\mathrm{EM}F_{x}\;\left(t\right)=-Av\frac{\partial B_{x}}{\partial x}$$

These expressions follow directly from the convective derivative of the magnetic flux under rigid translation and fixed loop orientation.

Rotational motion about the vertical (*Y*) axis was modeled by introducing a time‐dependent loop normal vector $\hat{n}$(*t*) defined by the rotation angle *φ*(*t*) = *ωt*, where *ω* is the angular velocity. The loop center position was assumed fixed in space during rotation. Its coordinates (*x*_0_, *z*_0_) correspond to the spatial location selected on the magnetic field map at thoracic height and represent the effective center of the equivalent loop area.

Under this condition, the magnetic field vector evaluated at the loop center, $\vec{B}$(*x*_0_, *z*_0_), is constant in time, and the induced EMF arises solely from the time variation of loop orientation:

(4) $$\mathrm{EM}F_{\mathrm{rot}}\;\left(t\right)=-A\frac{d}{dt}\left(\vec{B}\left(x_{0},z_{0}\right)\cdot \hat{n}\left(t\right)\right)$$

Peak EMF values and maximum effective field variation rates ($\frac{dB_{n}}{dt}$) were extracted from the simulated time histories for each motion condition. The adopted formulation preserves the exact Faraday‐law dependence while applying the standard locally uniform field approximation over the loop surface, commonly used in implant electromagnetic safety analyses.

The induced EMF values for representative high‐exposure scenarios were estimated by evaluating the maximum absolute spatial gradient of the magnetic field component normal to the loop for linear motions and the maximum perpendicular field magnitude for rotational motion over the full measurement grid.

Maximum effective temporal magnetic field variation rates were derived from peak EMF values as (*dB*/*dt*)max = EMFmax/*A*, consistent with Faraday's law under the locally uniform field approximation.

A reference velocity *v* of 1 m/s was adopted for linear motion (and *ω* = 1 rad/s for rotation) to provide normalized results. Since the induced EMF scales linearly with velocity, the reported values can be directly rescaled to represent different motion conditions. These values should therefore not be interpreted as worst‐case estimates.

### 2.1 Magnetic field measurement, spatial reconstruction, and coordinate system

The static magnetic field input to the computational framework was derived from experimental measurements performed in the MRI rooms using a tri‐axial Hall probe magnetometer (HP‐01, Narda Safety Test Solutions, Italy).^27^ The instrument allows independent acquisition of the orthogonal components of the magnetic field vector. In the present study, the components *Bx* and *Bz* were retained, as they represent the dominant contributions to magnetic flux variation for the motion scenarios considered.

Measurements were acquired over the region accessible to operators in proximity to the scanner gantry, focusing on the fringe‐field area relevant for occupational exposure. Field sampling was performed on horizontal planes (*x*–*z* planes) at thoracic height using a regular grid with 0.1 m spacing. The measurement domain extended from the bore entrance outward into the MRI room, covering the spatial region where typical worker movements occur.

The measured magnetic field data were expressed within a right‐handed Cartesian coordinate system defined with respect to the MRI scanner geometry. The origin of the coordinate system was set at the scanner isocenter, identified using the system positioning lasers. The *z*‐axis was aligned with the scanner bore and patient table direction (head–foot direction). The *x*‐axis was defined in the horizontal plane as the left–right direction, perpendicular to the bore axis. The *y*‐axis was defined as the vertical direction. Probe positioning during measurements was performed manually using a rigid support to ensure reproducibility of spatial coordinates and to minimize angular misalignment. The overall positioning uncertainty was estimated to be on the order of a few millimeters.

The discrete measurement dataset was interpolated onto a finer regular grid (0.01 m resolution) using cubic interpolation to enable stable numerical evaluation of spatial derivatives.^27^ Spatial gradients of the magnetic field components were then computed using central finite‐difference schemes. This processing provides a continuous representation of the magnetic field components over the region of interest, suitable for trajectory‐based EMF evaluation.

It should be noted that the field mapping is based on measurements performed on a single horizontal plane and therefore, does not provide full volumetric characterization of the magnetic field. Variations along the vertical direction (*y*‐axis) and full three‐dimensional vector field reconstruction were not directly measured. As a consequence, off‐axis directionality effects and local rotations of the magnetic field vector may not be fully captured by the present model.

Measurement uncertainty arises from a combination of instrumental accuracy (≈1%), probe positioning error, and interpolation and derivative estimation. Based on these contributions and on the variability observed in the spatial dataset, the overall uncertainty on derived field gradients is estimated to be on the order of 20%–30% in regions of high spatial variation.^27^

A comparison between the measured magnetic field distributions and the manufacturer‐provided *B*0 isogauss maps was previously performed in our earlier work.^27^ That analysis showed good agreement, especially along the *z*‐axis. It should also be noted that manufacturer‐provided isogauss maps are themselves approximate, as they are typically derived for idealized empty environments and do not account for site‐specific factors such as shielding structures or surrounding equipment. As a result, deviations between measured and reference maps are expected even under accurate measurement conditions.

### 2.2 Interactive simulation environment

An interactive simulation environment was developed in MATLAB to support scenario‐based evaluation of motion‐induced EMF. The tool integrates measured magnetic field maps with a graphical user interface that allows the operator to define motion conditions directly on the spatial field map.

The simulator displays the two‐dimensional magnetic field distribution in the *x*–*z* plane at thoracic height and enables graphical selection of spatial locations through mouse input. Users can select the initial position for motion trajectories or the fixed pivot point for rotational motion directly from the map representation of the MRI room layout. Linear motion scenarios can be defined by specifying movement direction and trajectory endpoints, while rotational scenarios are defined by selecting the fixed loop‐center location and rotation parameters.

User‐defined inputs include motion type (linear or rotational), direction of translation, walking velocity or angular velocity, and effective loop area. Based on these inputs, the simulator automatically generates the corresponding spatial trajectory or orientation time history and computes the induced EMF using the field interpolation and gradient‐based formulations described above.

The graphical interface also provides visual feedback of the selected trajectory, loop orientation, and locations of peak induced EMF along the motion path. This interactive framework enables rapid exploration of multiple exposure scenarios and supports safety assessment under user‐defined movement conditions.

### 2.3 Multi‐scanner environments

The computational framework was applied to two distinct MRI environments characterized by different nominal static field strengths: a clinical 3 T system and an ultra‐high‐field 7 T system. For each system, measured two‐dimensional maps of the static magnetic field components *Bx*(*x*,*z*) and *Bz*(*x*,*z*) at thoracic height were used as model inputs. The maps were obtained as previously described.^27^, ^28^ All preprocessing, interpolation, and gradient computations were performed independently for each scanner dataset using the same numerical pipeline, ensuring methodological consistency across field strengths. Motion‐induced EMF simulations, including linear translations parallel and perpendicular to the scanner axis as well as rotational motions, were then executed separately for each field environment using identical loop geometry and motion parameter settings unless otherwise specified. This dual‐environment modeling approach enables direct comparison of induced EMF levels and spatial distributions across different static field strengths and fringe‐field gradient structures, while maintaining identical geometric and computational assumptions.

An artificial intelligence‐based language model was used to assist with language editing and drafting support during manuscript preparation. All scientific content, modeling choices, data analysis, and interpretation of results were developed and verified by the authors.

## 3 RESULTS

### 3.1 Spatial field and gradient characterization

Measured static magnetic field maps acquired at thoracic height showed the expected spatial decay with increasing distance from the scanner bore for both MRI systems. The 3 T system exhibited steeper local spatial variations of the field components near the gantry region, while the 7 T system showed a broader spatial distribution with smoother decay patterns.

**FIGURE 1:**
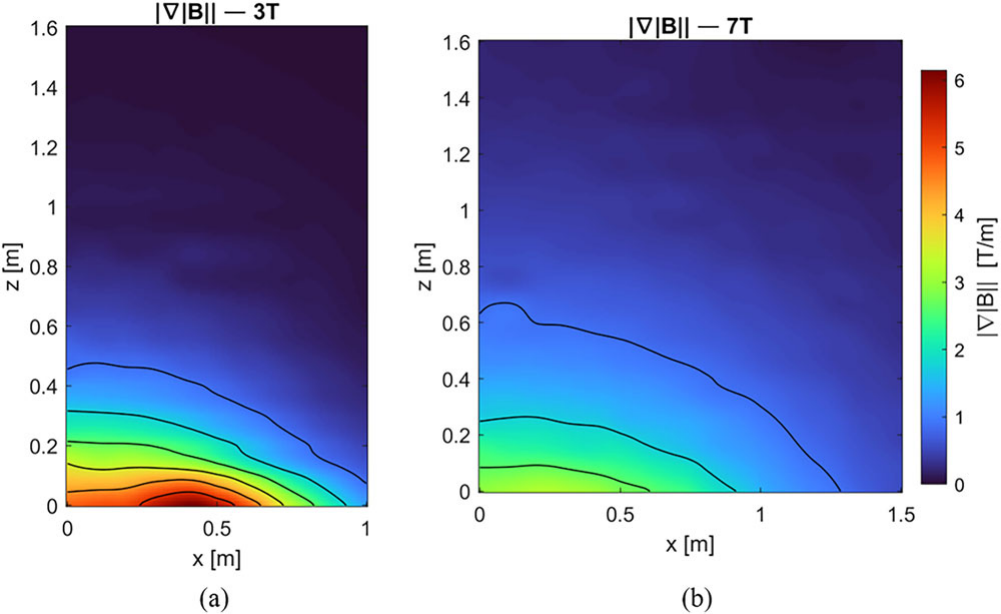
Spatial maps of the magnetic field magnitude gradient for (a) 3 T and (b) 7 T systems.

For visualization and interpretation purposes, spatial maps of the magnetic field magnitude gradient were computed from the interpolated component maps. The gradient magnitude distributions confirmed that the highest spatial variations are localized in the near‐bore fringe region for both scanners, with spatial extent and peak values differing between the two systems. A common color scale was used to enable direct visual comparison between scanners (Figure 1).

The spatial field gradients shown in Figure 1 represent values derived from measurements within a specific region of the MRI room at thoracic height. These values do not necessarily correspond to the maximum gradients achievable within the MRI system, which may occur in regions not covered by the measurement grid. Literature data indicate that higher peak gradients may be present depending on system design and measurement location.^29^

These gradient maps were used only to support physical interpretation of motion‐induced coupling, while EMF calculations were strictly based on the spatial derivatives of the magnetic field component normal to the loop surface, as defined in Methods.

### 3.2 Motion‐induced EMF—Linear translations

Simulated linear motion of the PM loop through the measured fringe field produced position‐dependent EMF values proportional to loop area, velocity, and local spatial gradient of the field component normal to the loop surface.

**FIGURE 2:**
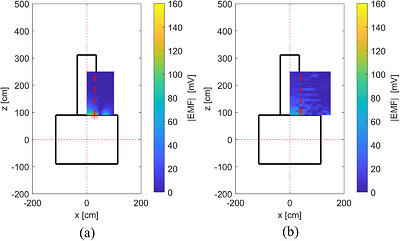
Peak induced EMF map for linear motion along the *z*‐axis. Results are shown for (a) 3 T and (b) 7 T systems under identical loop and motion parameters. The dashed line indicates the simulated walking path, and the marker identifies the location of maximum EMF along the trajectory. The black outline represents the MRI scanner and table footprint in top view. EMF, electromotive force; MRI, magnetic resonance imaging.

For identical motion parameters (loop area 0.0225 m^2^, velocity 1 m/s), peak induced EMF values differed between motion directions and scanner systems. For linear motion parallel to the scanner axis (*z*‐direction), the maximum EMF was (Figure 2):

- 3 T system: 159.1 mV
- 7 T system: 80.8 mV

**FIGURE 3:**
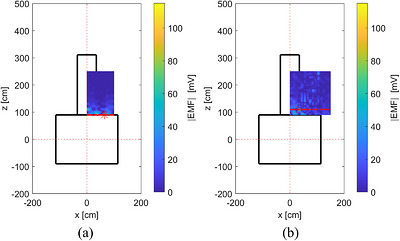
Peak induced EMF map for linear motion along the *x*‐axis. Results are shown for (a) 3 T and (b) 7 T systems under identical loop and motion parameters. The dashed line indicates the simulated walking path, and the marker identifies the location of maximum EMF along the trajectory. The black outline represents the MRI scanner and table footprint in top view. EMF, electromotive force; MRI, magnetic resonance imaging.

For linear motion perpendicular to the scanner axis (*x*‐direction), assuming torso orientation such that the loop plane rotated accordingly and the normal field component changed to *Bx*, the maximum EMF was (Figure 3):

- 3 T system: 113.5 mV
- 7 T system: 103.7 mV

In the 3T system, motion in the *z* direction yielded higher peak EMF values than motion in the *x* direction, which is consistent with the larger spatial gradients observed along the latter direction (max |*∂Bz*/*∂z*| = 7.07 T/m vs. max |*∂Bx*/*∂x*| = 5.04 T/m).

In the 7 T system, motion in the *x* direction yielded higher peak EMF values than motion in the *z* direction, which is consistent with the larger spatial gradients observed along the *z* direction (max |*∂Bz*/*∂z*| = 3.59 T/m vs. max |*∂Bx*/*∂x*| = 4.61 T/m).

Representative high‐exposure EMF locations were automatically identified over the spatial grid and were consistently found in regions close to the gantry where spatial field gradients are largest.

The spatial patterns observed in the EMF maps (Figures 2 and 3) reflect the local structure of the measured magnetic field components and their spatial derivatives. In particular, localized regions of elevated EMF correspond to areas where the spatial gradient of the relevant field component *(∂Bz*/*∂z* or *∂Bx*/*∂x*) exhibits rapid changes. These features are influenced by the non‐uniform fringe‐field distribution, which is shaped by the specific magnet design and shielding configuration of the scanner. In addition, interpolation of discrete measurement data may amplify small‐scale variations. As a result, the EMF maps should be interpreted as a spatially resolved representation of gradient‐driven induction effects rather than as smooth field distributions. Despite these local irregularities, the overall spatial trends, including the identification of high‐risk regions near the bore entrance and off‐axis locations, are robust and consistent with the underlying measured field gradients.

### 3.3 Motion‐induced EMF—rotational motion

**FIGURE 4:**
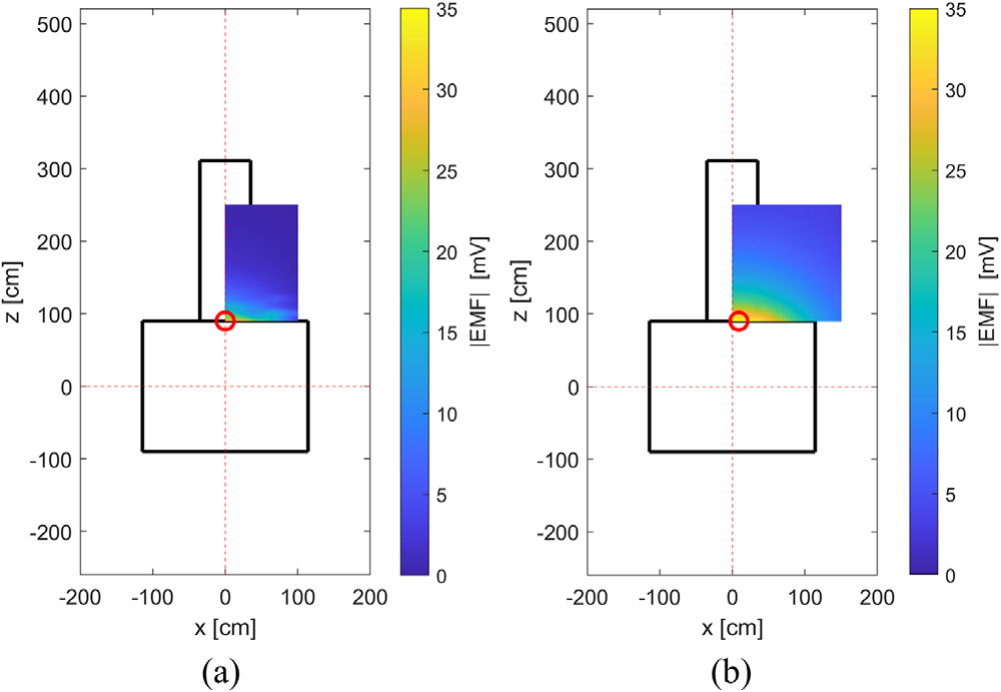
Peak induced EMF map for rotational motion. Results are shown for (a) 3 T and (b) 7 T systems under identical loop and motion parameters. The marker identifies the location of maximum EMF. The black outline represents the MRI scanner and table footprint in top view. EMF, electromotive force; MRI, magnetic resonance imaging.

For rotational motion of the torso around the vertical axis with a fixed loop center, EMF arises exclusively from the time variation of loop orientation relative to the static magnetic field vector. Using an angular velocity of 1 rad/s and an identical loop area, the peak EMF values were (Figure 4):

- 3 T system: 29.8 mV
- 7 T system: 35.5 mV

In contrast to the findings for linear motion, the rotational EMF did not scale directly with fringe gradient magnitude. Instead, it was found to depend primarily on the local vector field composition at the selected position. This finding thus validates the moderately elevated rotational maximum EMF observed in the 7 T system in comparison to the 3 T system.

Time histories of EMF during rotation showed sinusoidal behavior consistent with the theoretical dependence on the dot product between the magnetic field vector and the rotating loop normal.

### 3.4 Representative high‐exposure scenario spatial mapping

Table 1 summarizes the induced EMF for representative high‐exposure scenarios found under fixed motion parameters together with spatial locations for each motion type. The EMF values were localized in the high‐gradient fringe‐field regions in close proximity to the bore entrance. Representative high‐exposure scenarios coordinates were automatically extracted and overlaid on field and gradient maps to support traceability between spatial field structure and induced voltage maxima (Figures 2, 3, 4). Spatial EMF maps demonstrated strong anisotropy with respect to motion direction, confirming the importance of jointly modeling loop orientation and movement direction in exposure assessment.

**TABLE 1:** Representative high‐exposure EMF values and spatial coordinates for each motion type.

	EMF (mV) Linear path (along *z*), *v* = 1 m/s	EMF (mV) Linear path (along *x*), *v* = 1 m/s	EMF (mV) Rotation *θ* = 180°, w = 1 rad/s
*B*0 = 3 T	159.10	113.50	29.81
	@*x* = 29, *z* = 90 cm	@*x* = 65, *z* = 90 cm	@*x* = 0, *z* = 90 cm
*B*0 = 7 T	80.81	103.72	35.45
	@*x* = 40, *z* = 104 cm	@*x* = 35, *z* = 110 cm	@*x* = 9, *z* = 90 cm

Expressed as equivalent temporal magnetic field variation rates using *dB*/*dt* = EMF/*A*, the representative high‐exposure values correspond to approximately 5–7 T/s for linear motion in the 3 T environment and 3–5 T/s in the 7 T environment, while rotational motion produced values near 1–2 T/s in both systems. These values represent high‐end estimates within the analyzed motion scenarios and should not be interpreted as absolute upper bounds, as additional motion patterns and three‐dimensional field variations were not explored.

### 3.5 Cross‐scanner comparison under matched motion conditions

Direct comparison between the 3 and 7 T systems under identical motion parameters and loop geometry showed that higher nominal field strength does not necessarily produce higher motion‐induced EMF. Instead, induced voltage levels were primarily determined by local fringe‐field spatial gradients and field vector orientation.

**FIGURE 5:**
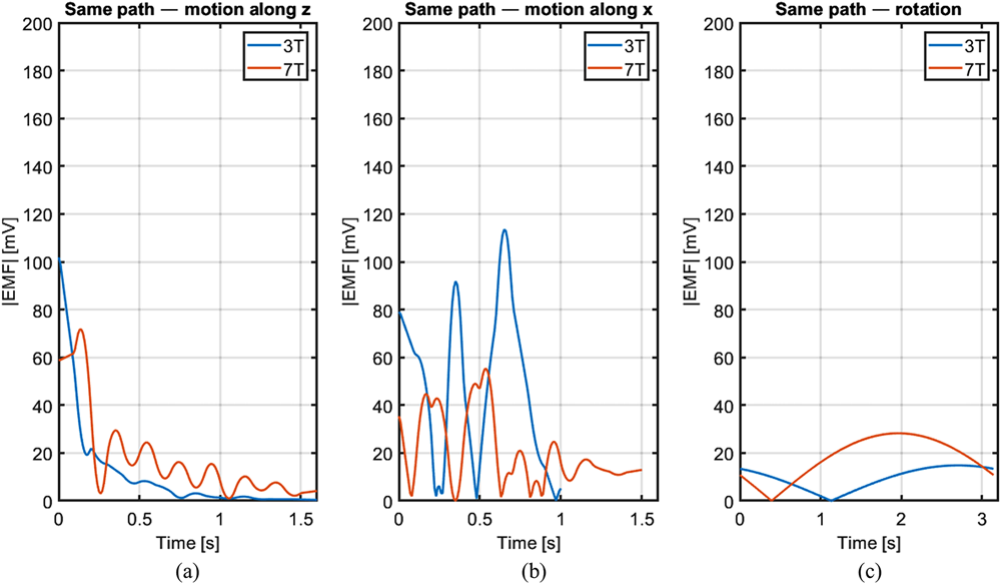
|EMF| value along time for same paths in 3 and 7 T systems: (a) motion along *z*, (b) motion along *x*, and (c) rotation. EMF, electromotive force.

For the measured datasets analyzed here, the 3 T scanner produced higher peak EMF values for linear translations in both principal directions. Conversely, the rotational peak EMF value in the 7 T case was higher with respect to the 3 T case at the tested location (see Figure 5).

This behavior is consistent with the theoretical dependence of translational induction on spatial field gradients and of rotational induction on local field magnitude. The 7 T system that was analyzed exhibited more confined fringe fields and lower spatial gradients in the accessible regions that were considered. This resulted in reduced motion‐induced EMF despite the higher nominal static field strength. Despite the implementation of identical rotational kinematics, the temporal progression of the induced EMF, as shown in Figure 5c, exhibited variations between the two MRI systems. This discrepancy can be attributed to the distinct local vector composition of the magnetic field inherent in each system. The rotational EMF is determined by the projection of the field vector onto the time‐varying loop normal, resulting in scanner‐specific phase and amplitude characteristics.

## 4 DISCUSSION

The present results demonstrate that motion‐induced EMF in a PM‐equivalent loop inside the MRI environment is strongly dependent on both motion type and local magnetic field spatial structure. In the 7 T scenario, linear motion perpendicular to the patient table (*x*‐direction) produced the highest peak EMF values, followed by linear motion parallel to the table (*z*‐direction). In the 3 T scenario, instead, linear motion parallel to the patient table (*z*‐direction) produced the highest peak EMF values, followed by linear motion perpendicular to the table (*x*‐direction). In both scenarios, rotational motion around the vertical axis generated substantially lower EMF amplitudes under the same loop area and motion speed assumptions.

A key finding is that the 7 T system did not systematically produce higher induced EMF compared to the 3 T system. For linear motion along the *z*‐axis, peak EMF values were approximately a factor of two lower at 7 than at 3 T for the investigated trajectories, whereas rotational motion produced comparable magnitudes between scanners. This confirms that motion‐induced EMF is governed primarily by local spatial gradients and vector field composition rather than by nominal *B*0 field strength alone. Regions with stronger fringe‐field spatial variation in the 3 T installation produced larger effective *dB*/*ds* values than the corresponding accessible regions in the 7 T room layout considered here.^30^ This finding aligns with the established literature on the subject. For instance, earlier research has documented upper‐bound values of approximately 19, 17, and 16 T/m for 1.5, 3, and 7 T clinical systems, respectively.^29^ This observation highlights that motion‐induced EMF may be comparable or even higher in lower‐field systems depending on magnet design and shielding configuration. Therefore, the assessment of motion‐induced EMF should be based on system‐specific spatial field distributions rather than nominal field strength alone. The measurement‐based framework adopted in this study is inherently general and can be applied to different MRI systems, including 1.5 T scanners, provided that the corresponding spatial field maps are available.

The directional dependence observed between *x* and *z* translations is consistent with the theoretical formulation derived from Faraday's law. For a loop lying in the vertical plane, the effective flux‐driving component is the magnetic field component normal to the loop surface. Consequently, motion along *z* couples to *∂Bz*/*∂z*, whereas motion along *x* couples to *∂Bx*/*∂x* when the operator orientation changes and the loop plane rotates accordingly. In the 7 T scenario, higher EMF observed for *x*‐directed motion indicates steeper lateral field gradients in the measured maps compared to longitudinal gradients along the table axis.

Rotational motion produced lower peak EMF values because induction arises from orientation change rather than spatial gradient traversal. In this case, EMF scales with the angular velocity and with the magnitude of the local in‐plane magnetic field vector. The measured values confirm the expected proportionality to |$\vec{B}$| at the rotation center and show reduced sensitivity to localized gradient hotspots.

It is worth noting that time‐varying magnetic fields generated by imaging gradient switching can induce EMFs that are typically higher than those arising from motion in the static magnetic field. However, gradient‐induced exposure is inherent to MRI operation, is well controlled, and is explicitly considered in device safety standards. In contrast, motion‐induced EMF depends on subject movement within the spatially varying *B*0 field and may occur outside active imaging conditions, such as during patient handling. Therefore, the two mechanisms should be regarded as complementary, and the assessment of motion‐induced EMF provides additional insight into exposure conditions not fully captured by standard MRI safety evaluations.

In this study, the PM/ICD lead system was represented using an equivalent single‐loop model with effective area, a commonly adopted abstraction in implant electromagnetic safety analyses.^23^ This approach captures the dominant magnetic flux coupling mechanism while neglecting fine geometrical details of the actual lead routing and return paths. The loop area parameter can be interpreted as an effective coupling area and can be adjusted to represent different implant configurations.

The magnetic field was assumed locally uniform over the loop surface when evaluating magnetic flux. Under this assumption, the surface integral of the magnetic field reduces to the product of loop area and the magnetic field component normal to the loop plane.^23^ This approximation is valid when the spatial variation of the field across the loop dimensions is small relative to the absolute field level. For typical PM/ICD loop dimensions (order of 5–15 cm) and MRI fringe‐field spatial gradients, this condition is generally satisfied, although localized high‐gradient regions may introduce additional second‐order corrections.

The field input consisted of two‐dimensional maps evaluated at thoracic height. Vertical field variation and fully three‐dimensional spatial effects were therefore not explicitly modeled. While this approach is appropriate for planar motion scenarios at fixed height, it does not capture potential variations along the vertical direction or more complex spatial field structures. As a result, the induced EMF may be either underestimated or overestimated depending on the specific motion trajectory and local field configuration. Consequently, the present results should not be interpreted as conservative estimates in a general sense, but rather as scenario‐specific evaluations based on the available field measurements. Despite these limitations, the adopted approach enables a physically consistent and spatially resolved estimation of the magnetic field components and their gradients in the region of interest. This provides a first‐order approximation of the vector magnetic field distribution, which is sufficient to support motion‐induced EMF calculations and to verify the internal consistency of the measured field maps.

The contribution of tissue conductivity, lead impedance, and device‐specific circuit responses was not modeled in the present formulation. This approach is consistent with the method adopted by the current technical standards (ISO 14117, EN 50527‐1, and EN 50527‐2‐1) for the calculation of the induced voltage from low‐frequency magnetic fields. Consequently, the model estimates open‐circuit induced EMF rather than induced current. Conversion to induced current would require coupling with an electrical model of the lead–device system.

Finally, motion was modeled as rigid‐body translation or rotation at constant velocity or angular velocity. More complex biomechanical motion patterns could be incorporated in future extensions by prescribing time‐dependent trajectories and orientations within the same computational framework.

From a device safety perspective, the computed peak EMF values fall in the tens to hundreds of millivolt range for the assumed loop area (225 cm^2^) and motion parameters (1 m/s, 1 rad/s). These amplitudes are comparable to or above typical sensing thresholds of cardiac implantable electronic devices, indicating that motion‐induced voltages in fringe‐field regions may represent a non‐negligible coupling mechanism, particularly during brisk operator movement near the bore entrance. While modern PM leads include filtering and protection circuitry, these results support the need for motion‐aware exposure assessment rather than static field criteria alone.

The resulting EMF cannot be immediately compared to the programmed sensitivity threshold of the PM/ICD^31^, ^32^ (typically 2 mV in unipolar setting, 0.3 mV in bipolar setting), since it is defined for a standardized stimulus having higher frequency components than the low‐frequency voltage signal induced by the movement inside the MRI magnetic field (around 1 Hz^33^). It is likewise difficult to directly compare the computed EMF with the immunity levels that PM/ICD systems must comply with according to international standards for market approval, as these immunity tests are frequency‑dependent and do not consider signals below 16.6 Hz. In particular, ISO 14117 defines conducted immunity tests starting from 16.6 Hz, with a test voltage level of 2 mV (peak‐to‐peak) that remains constant up to 1 kHz. Although this test level applies to higher frequencies and to standardized waveform conditions that differ substantially from the low‑frequency, motion‑induced EMF investigated here, it nonetheless represents the lowest available reference level specified by current standards. For this reason, the 2‐mV immunity level can only be regarded as a first‑order benchmark to contextualize the magnitude of the predicted EMF and to support a preliminary, screening‑level risk assessment, while acknowledging that a formal comparison is not possible and that device‑specific responses at sub‑16 Hz frequencies are not explicitly addressed by existing immunity standards.

Moreover, the induction area A used here has to be chosen according to the sensing modality of the cardiac stimulator: in bipolar sensing, the induction area is reduced to a factor of about 17 compared to the unipolar sensing.^34^ In any case, the EMF predicted by the presented framework is far above both the typical sensitivity settings of PM/ICD systems and the immunity test levels required by international standards in the low‑frequency range (2 mV). This confirms that motion within a highly spatially varying static magnetic field, such as that found in the MRI environment, can induce device malfunction, resulting in oversensing episodes. These findings are consistent with experimental in‐vitro results reported in the literature.^23^, ^24^

Beyond the quantitative results, an important practical advantage of the proposed framework is its computational simplicity and operational accessibility. The simulator is based on directly measured magnetic field maps and closed‐form Faraday‐law relationships, and therefore does not require full‐wave electromagnetic solvers, anatomical models, or device‐specific proprietary information.

The interactive implementation further allows non‐expert users to select starting positions, motion types, and trajectories through a graphical interface, immediately obtaining time‐resolved EMF estimates and peak values. This makes the tool suitable not only for research analysis but also as a practical screening and comparative risk‐assessment instrument for MRI facilities, supporting identification of higher‐risk regions and motion patterns without the need for complex numerical infrastructure.

Because the method is map‐based and scanner‐specific, it naturally supports site‐dependent evaluation and can be updated whenever new field measurements or room configurations become available. This enables motion‐specific, position‐resolved EMF estimation and identification of representative high‐exposure locations and trajectories within a given MRI room. Such a tool can support site‐specific safety evaluation and protocol optimization.

A possible methodological refinement concerns the approximation of magnetic flux through the equivalent PM/ICD induction loop. Future work could extend the framework toward surface‐integrated flux evaluation by reconstructing the magnetic field distribution over the loop plane using multi‐height field measurements and three‐dimensional interpolation.^27^ Such an approach would enable direct numerical flux integration and explicit estimation of spatial non‐uniformity effects across the loop area. However, this refinement would require denser volumetric field mapping to ensure that interpolation uncertainty remains smaller than the correction being estimated. For this reason, the present center‐field approximation represents a robust first‐order model for motion‐induced EMF risk screening, while higher‐order spatial integration can be considered a second‐stage refinement when high‐resolution 3D field data are available.

A further relevant development would be the integration of the proposed simulation framework with controlled phantom‐based experiments on PM/ICD systems, similar to those previously reported in the literature using conductive torso phantoms and instrumented leads.^23^ In such a combined approach, the motion‐dependent EMF predicted by our model for specific trajectories and room locations could be reproduced experimentally by controlled movement of the implant–lead system within measured MRI fringe fields. This would enable direct correlation between estimated induced EMF and observed device behavior, such as sensing disturbances or inappropriate event detection. The coupling of map‐based EMF prediction with phantom AIMD testing would provide a practical validation pathway and support translation of the present method from exposure screening toward device‐level risk characterization.

The present study focuses on deterministic motion trajectories to enable a controlled and physically interpretable analysis of motion‐induced EMF in MRI environments. While this approach allows for a clear identification of the relationship between spatial field gradients, motion characteristics, and induced EMF, it does not capture the full variability of real‐world operator movements. In practical scenarios, worker motion in the vicinity of MRI systems is inherently variable and not predefined. Therefore, an extension of the present framework toward the analysis of trajectory ensembles and probabilistic motion models would represent a valuable development. Such an approach would enable the definition of exposure envelopes and statistical descriptors (e.g., percentiles of induced EMF) that are more representative of realistic occupational conditions. The current model provides a suitable foundation for this extension, as it allows rapid evaluation of EMF along arbitrary trajectories and can be readily integrated with stochastic or data‐driven motion inputs.

In this study, the frontal region was prioritized as it represents the primary interaction zone for operators; however, extension to lateral and posterior regions will require dedicated measurement campaigns. Moreover, realistic motions such as bending around the hip involve coupled translation and rotation and would require full three‐dimensional magnetic field mapping for accurate modeling.

Furthermore, it is important to emphasize that the calculation of motion‐induced EMF represents an intermediate step in the overall assessment of safety for PM‐carrying individuals in MRI environments. While EMF quantifies the physical driving mechanism for potential device interference, the actual risk depends on additional factors, including lead configuration, device‐specific susceptibility, and filtering characteristics of the implant system. Therefore, the results presented here should be interpreted as a quantitative estimation of exposure at the electromagnetic level, which can serve as input to higher‐level models addressing device response and clinical risk. Future work will aim to couple the present EMF estimation framework with probabilistic trajectory modeling and device‐level response models, enabling a more comprehensive and application‐oriented assessment of occupational safety in MRI settings.

## 5 CONCLUSIONS

This work presents a computational framework for estimating motion‐induced EMF in PM/ICD‐equivalent lead loops within MRI fringe‐field environments. The results show that induced EMF depends mainly on motion type, trajectory, and local magnetic field spatial gradients. It does not rely solely on nominal scanner field strength. The proposed approach combines measured field maps with induction models and interactive motion definition. This combination enables position‐resolved and trajectory‐specific EMF estimation with minimal computational complexity.

The method is scanner‐specific, transparent, and easy to implement. It can serve as a practical screening and comparative risk‐assessment tool for MRI facilities. This supports identification of higher‐risk motion patterns and spatial regions for AIMD carriers and exposed operators. The framework is based on a first‐order equivalent‐loop approximation, but is readily extendable toward higher‐resolution field integration and experimental phantom validation. These characteristics make the tool a useful bridge between theoretical induction modeling and operational MRI safety evaluation in modern high‐field installations.

## CONFLICT OF INTEREST STATEMENT

The authors declare no conflicts of interest.
